# Genetic Background of Medication-Related Osteonecrosis of the Jaw: Current Evidence and Future Perspectives

**DOI:** 10.3390/ijms251910488

**Published:** 2024-09-29

**Authors:** Bence Bojtor, Bernadett Balla, Mihaly Vaszilko, Szofia Szentpeteri, Zsuzsanna Putz, Janos P. Kosa, Peter Lakatos

**Affiliations:** 1 Department of Internal Medicine and Oncology, Semmelweis University, 1083 Budapest, Hungary; bojtor.bence@stud.semmelweis.hu (B.B.); putz.zsuzsanna@med.semmelweis-univ.hu (Z.P.); kosa.janos@semmelweis.hu (J.P.K.); 2 Hungarian Research Network SE-ENDOMOLPAT Research Group, 1085 Budapest, Hungary; balla.bernadett@semmelweis.hu; 3 Department of Oro-Maxillofacial Surgery and Stomatology, Semmelweis University, 1085 Budapest, Hungary; vaszilko.mihaly.tamas@semmelweis.hu (M.V.); szentpeteri.szofia.katalin@semmelweis.hu (S.S.)

**Keywords:** medication-related osteonecrosis of the jaw, genetic background, single nucleotide polymorphism, gene expression, microRNA

## Abstract

Medication-related osteonecrosis of the jaw (MRONJ) is a rare side effect of antiresorptive drugs that significantly hinders the quality of life of affected patients. The disease develops in the presence of a combination of factors. Important pathogenetic factors include inflammation, inhibition of bone remodeling, or genetic predisposition. Since the first description of this rare side effect in 2003, a growing body of data has suggested a possible role for genetic factors in the disease. Several genes have been suggested to play an important role in the pathogenesis of MRONJ such as *SIRT1*, *VEGFA*, and *CYP2C8*. With the development of molecular biology, newer methods such as miRNA and gene expression studies have been introduced in MRONJ, in addition to methods that can examine the base sequence of the DNA. Describing the complex genetic background of MRONJ can help further understand its pathophysiology as well as identify new therapeutic targets to better manage this adverse drug reaction.

## 1. Introduction

Osteonecrosis or avascular necrosis is a pathological condition defined by the death of bone tissue due to the disruption in blood supply. It can affect various areas such as the femoral head, humeral head, spine, or jaw (osteonecrosis of the jaw; ONJ). ONJ is mainly caused by various medications (MRONJ), radiation therapy, and trauma to the jaw [1]. Medication-related osteonecrosis of the jaw (MRONJ) is a significant adverse effect of mainly antiresorptive drugs [1]. Antiresorptive agents such as bisphosphonates (BPs) or denosumab inhibit bone resorption by reducing the activity of osteoclast cells. Bisphosphonates bind to hydroxyapatite in bone and inhibit osteoclast function as well as induce apoptosis in osteoclast cells [2]. Denosumab is a monoclonal antibody that targets the receptor activator of nuclear factor kappa-B ligand (RANKL), a molecule essential for osteoclast function [3]. These agents are effective in the treatment of osteoporosis and in reducing the risk of skeletal-related events (SREs) (e.g., hypercalcemia, pathologic fracture, spinal cord compression, palliative radiotherapy to bone) in cancer patients [4,5]. The first case of bisphosphonate-related osteonecrosis of the jaw (BRONJ) was described more than twenty years ago, in 2003 [6]. Since that time, several other agents have been linked to similar maxillary and mandibular osteonecrotic conditions. Due to these new findings, the American Association of Oral and Maxillofacial Surgeons updated the terminology from BRONJ to MRONJ in 2014 [7]. Based on the agent causing the osteonecrosis, MRONJ cases can be divided into two distinct groups. These groups are bisphosphonate-related osteonecrosis of the jaw (BRONJ) and non-bisphosphonate-related osteonecrosis of the jaw (non-BRONJ). Non-BRONJ instances are caused by numerous drugs such as the anti-receptor activator of nuclear factor kappa-Β ligand (anti-RANKL) antibody denosumab, an inhibitor of mammalian target of rapamycin (mTOR) everolimus, tyrosine kinase inhibitors, or antiangiogenic drugs (e.g., bevacizumab) [8]. Evidently, MRONJ is a heterogeneous disease, as several drugs and diverse pathophysiological factors contribute to its development. This heterogeneity might hinder the identification of universal genetic factors, as distinct genetic factors may be implicated in different cases.

MRONJ is defined as bone exposed or accessible through an extra or intraoral fistula in the maxillofacial region that has persisted for more than 8 weeks, current or previous treatment with antiresorptive agents alone or in combination with other drugs (e.g., antiangiogenic agents, immune modulators), and no history of metastatic disease or radiation exposure to the jaw [1]. MRONJ incidence is approximately a magnitude higher in cancer patients (0–18 percent) compared with the incidence in individuals treated with osteoporosis (0.02–0.3 percent) [1].

Affected patients might experience symptoms including odontalgia, dull pain in the jaw, intraoral and extraoral swelling, sinus pain, purulent discharge, and exposed and necrotic bone [1,9]. Due to these symptoms, predominantly the pain associated with the disease, MRONJ significantly hinders the quality of life of the affected patient [10]. Moreover, a more severe clinical stage of the disease is associated with even worse quality of life (QoL) indices [11]. A recent study showed that improvement in disease status potentially predicts the improvement in QoL indices [12].

The exact pathomechanism of MRONJ is still not clear. Several factors are thought to play a role in the disease. Bone remodeling imbalance, the inhibition of angiogenesis, immune system dysfunction, inflammation, and soft tissue toxicity might all be important factors in the development of the disease [13]. Risk factors include local factors such as dentoalveolar operations and systemic factors like diabetes mellitus or corticosteroid therapy [1]. Aside from these mechanisms, certain genetic factors, mainly SNPs, are also reported to play a consequential role in the development of MRONJ [14].

Single nucleotide polymorphisms (SNPs) in various genes have been investigated in MRONJ, primarily through candidate gene approaches, whole exome sequencing, and genome-wide association studies. The identified genes predominantly relate to bone metabolism, immune system function, or inflammatory processes. While disease-associated polymorphisms might have diagnostic value in the future, they also enhance our understanding of the pathomechanism of MRONJ.

Some studies have also aimed to discover the possible role of microRNAs in the pathophysiology of the disease [15,16]. Several microRNAs play an important role in bone biology [17], thus a better understanding of the microRNA landscape in MRONJ would help us further unveil the pathophysiology of the disease as well as help detect new therapeutic strategies.

Aside from detecting SNPs and microRNAs, gene expression studies have also been conducted in MRONJ [18,19]. Identifying differently expressed genes (DEGs) in tissues associated with the condition can help further understand the pathophysiology as well as detect novel therapeutic targets and biomarkers. 

This paper aimed to describe the genetic factors associated with MRONJ susceptibility and identify potential avenues for progress in this scientific field.

## 2. Indications for Antiresorptive Therapy

Antiresorptive agents such as BPs (e.g., zoledronic acid, alendronate, ibandronate, risedronate) and denosumab are primarily used to treat osteoporosis, the most common metabolic bone disease [20]. Both BPs and denosumab are highly effective in the treatment of primary (e.g., postmenopausal osteoporosis) and secondary osteoporosis (e.g., glucocorticoid-induced osteoporosis) as well as increasing the bone mineral density and lowering fracture risk [4,21]. BPs are also the first-line therapeutic choice in Paget’s disease of the bone or osteitis deformans, another relatively common metabolic bone disease [22]. Moreover, antiresorptive therapy is also crucial in the management of SREs in patients with multiple myeloma (MM) and bone metastases. BPs significantly decrease skeletal morbidity and also effectively delay the first SRE [5]. Denosumab was detected to be even more effective in reducing the risk of SREs when compared to BPs [4]. Furthermore, denosumab also improved the pain outcomes and lowered the opioid needs of patients [4]. BPs are also the mainstay of treatment in osteogenesis imperfecta (OI), an inherited skeletal dysplasia [23]. The use of BPs is beneficial in OI, as they increase bone mass as well as reduce fracture incidence [23]. BPs are also used, alongside parathyroid hormone analogs, in the treatment of fibrous dysplasia (FD) [24]. FD is an uncommon monostotic or polyostotic fibro-osseous bone lesion, with the potential for malignant transformation, in which BPs are used to maintain bone density [24].

## 3. The Pathophysiology of MRONJ 

Since the first case was reported in 2003 by Marx et al. [6], the pathomechanism of MRONJ has still not been exactly described. There are several proven factors contributing to the condition, however, none of them can describe the disease alone.

Bone remodeling inhibition is recognized as one of the key hypotheses of MRONJ pathophysiology [1]. Both BPs and denosumab (DMB) hinder bone remodeling by inhibiting osteoclast function, which is supported by the histological assessment of ONJ animal models [25]. Zoledronate-treated animals displayed atypical osteoclast cells, detached from the bone surface at the osteonecrosis site [25]. The parathyroid hormone (PTH) also rescued necrotic lesions and facilitated wound healing in a rat MRONJ model [26]. As PTH promotes bone remodeling, this further validates bone remodeling inhibition as an important factor in the development of the disease [27].

BPs, mainly zoledronate, have a direct cytotoxic effect on human gingival fibroblasts [28]. Zoledronate might hinder wound healing by inhibiting gingival fibroblast cell proliferation and migration [28,29]. These deficits in fibroblast function can attenuate MRONJ development. Despite having no cytotoxic effect on fibroblasts, DMB still results in an incidence similar to that of BPs [30]. Thus, direct soft tissue toxicity might be less important in the pathogenesis of the condition.

Local immune dysfunction has also been reported as an important factor in the development of MRONJ [31]. It is well-known that patients with immune disorders (e.g., diabetes, autoimmune disorders) have higher chances of MRONJ [1]. BPs mainly affect the innate immune system such as the dendritic cells, macrophages, and neutrophil granulocytes [31]. Soft tissue toxicity leads to easier infection of the gingival mucosa, while dysfunction of the innate immune system prolongs the infection and inflammation [31].

Inflammation is also thought to be a key factor in the development of the disease [32]. An animal model of the disease demonstrated that MRONJ susceptibility was increased in periradicular disease after tooth extraction [25]. In another study, the extraction of healthy teeth did not increase the chance of developing the condition, while on the other hand, preexisting periapicular disease increased the osteonecrotic susceptibility [33]. Furthermore, Nakamura et al. detected that MRONJ was more likely to develop from teeth with local infections [34].

**Figure 1:**
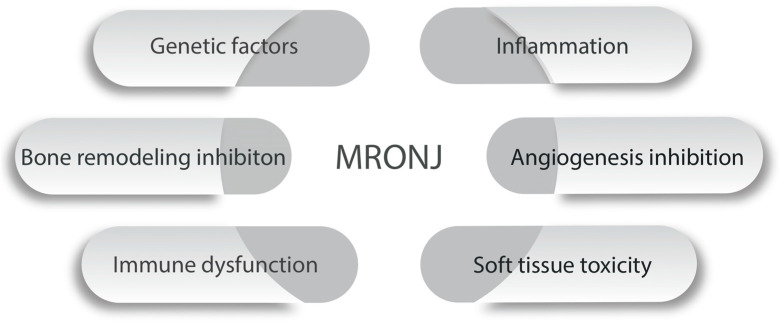
Main pathophysiological factors of MRONJ.

BPs have been demonstrated to inhibit angiogenetic pathways in multiple studies [35,36]. Moreover, locally delivered VEGF into healing extraction sockets lowered the MRONJ incidence through a pro-angiogenetic mechanism [37]. At the margins of osteonecrotic lesions, microvascular changes such as hypo-vascularized edematous areas have been detected [38]. These results suggest that angiogenesis inhibition might also be a contributing factor to MRONJ development. Figure 1 displays the most important factors contributing to the development of MRONJ.

## 4. Single Nucleotide Polymorphisms (SNPs) in MRONJ

### 4.1. CYP2C8

One of the most researched genes in the context of MRONJ is the *CYP2C8* gene, which encodes the cytochrome P4502C8 (CYP2C8) protein, a key member of the CYP2C family [39]. The cytochrome P450 proteins function as important enzymes in the oxidative metabolism of several drugs (e.g., paclitaxel, rosiglitazone, amodiaquine) [39]. Even though BPs are not directly metabolized by CYP2C8, this enzyme might have a functional role in periodontal fibroblast cells both in the metabolism of the drug and reactive oxygen species (ROS) [40]. Yamoune et al. found that zoledronic acid increased the ROS activity for certain genetic variants of the CYP2C8 protein [40]. Increased oxidative stress has been found to be a risk factor for developing MRONJ due to its ability to suppress bone turnover [41]. Moreover, CYP2C8 also alters the metabolic pathway involved in cholesterol production, which is crucial for osteoblast and osteoclast function [42,43].

In 2008, Sarasquete et al. conducted a genome-wide single nucleotide polymorphism analysis in MM patients [44]. In this study, 22 BRONJ cases and 65 matched BRONJ-free cases were compared. Four intronic SNPs, all in the *CYP2C8* gene, were found to be associated with BRONJ, distinctly rs1934951, which is located at intron 8 in the *CYP2C8* gene [44]. Some studies involving MM and prostate cancer patients were unable to detect an association between the genotype of rs1934951 and BRONJ [45,46,47]. Interestingly, Balla et al. found a significant correlation between rs1934951 and the anatomic localization of BRONJ [48]. In this study, AG carriers had a significantly higher chance of developing ONJ in mandibular localization [46]. Interestingly, Kastritis et al. found that the high-risk allele of rs1934951 SNP was associated with the earlier development of BRONJ [49]. Thus, carriers of the high-risk allele of rs1934951 are more likely to develop BRONJ earlier compared to non-high-risk allele carrier patients [49].

Due to the contradictory results regarding the relevance of *CYP2C8* rs1934951 in BRONJ, Zhong et al. performed a meta-analysis to determine the association between the polymorphism and BRONJ susceptibility [50]. In the pooled analysis, there were 126 cases and 453 controls. This meta-analysis did not detect a significant correlation between the genotype of rs1934951 and BRONJ. However, a subgroup analysis only considering MM patients showed a significant correlation between the disease and the SNP [50].

Overall, there is still not enough evidence to conclude on the significance of the *CYP2C8* gene in MRONJ. Studies with higher case numbers and matched control groups are needed to evaluate the role of *CYP2C8* in the disease.

### 4.2. SIRT1

The *SIRT1* gene encodes SIRT1, a member of the sirtuin (SIRT) protein family. Sirtuins are nicotine adenine dinucleotide^+^ (NAD^+^) dependent histone deacetylases that are involved in several crucial biological processes (e.g., inflammation, apoptosis, cell metabolism, cell proliferation) by epigenetically regulating numerous important genes in various biological pathways [51]. *SIRT1* is involved in several age-related diseases such as different cancers, cardiac hypertrophy, and Alzheimer’s disease [51]. SIRT1 is also an important regulator in bone homeostasis, primarily promoting bone formation through various biological processes [52]. These include activating the Wnt/β-catenin pathway by preventing β-catenin degradation, inhibiting osteoblast apoptosis, and also promoting autophagy to resist external cellular stress [52].

A study found that in both ovariectomized female mice and aged male mice (models for post-menopausal and age-related osteoporosis, respectively), a significant improvement in bone mass could be achieved by administering a SIRT1 agonist [53]. Moreover, a randomized placebo-controlled trial detected that a first-generation SIRT1 agonist, resveratrol, increased the bone mineral density (BMD) in obese men [54]. Another study found that oncologic doses of zoledronic acid (ZA) caused SIRT1-dependent inflammation in human oral keratinocytes (HOKs), which is recognized as a major risk factor for mucosal nonunion in MRONJ [55]. Additionally, menaquinone-4 was found to prevent MRONJ by alleviating osteoblast apoptosis via a SIRT1-dependent pathway [56].

In 2018, an exome-wide association analysis detected an association between MRONJ and two SNPs on chromosome 10 in the *SIRT1* and *HERC4* genes [57]. In this study, *SIRT1* rs7896005, an intronic SNP, was associated with lower odds of developing the condition. Furthermore, in silico analysis revealed that this SNP was an expression quantitative trait locus (eQTL) of the *SIRT1* gene in whole blood, with the minor A allele increasing the gene expression, resulting in a lower risk for MRONJ [57]. In a follow-up study, Yang et al. aimed to identify causal SNPs explaining the association between rs7896005 and MRONJ susceptibility [58]. They found that rs932658, an SNP in the promoter region of the *SIRT1* gene, was causally related to susceptibility to the disease [58]. In this study, a higher expression of the *SIRT1* gene was detected with the minor A allele of the rs932658 SNP, which might act as a protective factor in MRONJ [58]. In a recent paper, Bojtor et al. further validated the association between rs932658 and disease incidence in a retrospective study involving 63 osteoporosis and cancer MRONJ patients [59]. Furthermore, patients with rs932658 minor allele A had better healing tendencies from MRONJ compared to patients with other allelic variants [59].

In conclusion, *SIRT1* rs932658 is a promising SNP as a novel diagnostic tool to analyze disease susceptibility in patients at risk. Moreover, further validation of this polymorphism causal relationship with the disease might help better understand the pathomechanism of the disease as well as help to determine new SIRT1-associated drug targets in MRONJ therapy.

### 4.3. ESR1 and CYP19A1

Estrogen receptor and aromatase enzyme coding genes have also been researched in MRONJ, as estrogens are recognized as key regulators of bone remodeling in both sexes [55]. The effect of estrogens on bone cells is mediated by two nuclear receptors: ERα and ERß, encoded by the *esr1* and *esr2* genes, respectively [60]. Estrogens play a crucial role in maintaining BMD and mass via several pathways, which include repressing pro-osteoclastic cytokines (e.g., IL-1, IL-6) [61,62], anti-apoptotic effects in osteoblasts [63], and increasing the transcription of osteoprotegerin (OPG) [64]. Furthermore, both the *ESR1* and *ESR2* polymorphisms have been shown to affect the bone mass in humans [60]. Interestingly, ESR1 also regulates insulin-like growth factor-1 (IGF-1) activation, which is involved in re-epithelization and wound healing processes [65,66]. Based on these findings, *ESR1* polymorphisms can affect MRONJ susceptibility through both bone-specific and wound-healing mechanisms.

A study from 2023 involving 125 bisphosphonate-taking postmenopausal women found two SNPs (rs4870056 and rs78177662) in the *ESR1* gene that were significantly associated with MRONJ occurrence [67]. Both variants increased the risk of the condition by approximately 2.5-fold [67]. Rs4870056 is located in an intronic region, and has a strong link with another *ESR1* SNP, rs2234693 (Pvull) [67]. *ESR1* Pvull (rs2234693) is one of the most researched polymorphisms in the *ESR1* gene, which is associated with numerous diseases (e.g., cardiovascular diseases, breast cancer) [68,69], and might also have an effect on BMD [70].

Aromatase, encoded by the *CYP19A1* gene, is part of the cytochrome P450 enzyme family, responsible for transforming androgen precursors to estrogenic compounds [71]. The aromatase enzyme is expressed in several extraglandular sites such as adipose tissue or bone [72]. Moreover, aromatase activity is necessary for longitudinal bone growth and may significantly affect bone loss [71].

Aromatase polymorphism g.132810C>T was found to be significantly associated with BRONJ development in a study by [73], where patients with a TT homozygous genotype had a twofold higher risk for the development of BRONJ [73]. Interestingly, this genotype is associated with higher levels of local estrogens [74]. According to the author’s theory, locally higher estrogen levels and BPs inhibit bone remodeling more than BPs alone, leading to an increased risk for BRONJ [73].

To conclude, genes associated with estrogen pathways present a promising possibility for understanding and predicting MRONJ better in the future. However, there are only limited data available on these genes, so further studies are very much needed.

### 4.4. Genes Associated with Osteoclast Function and Bone Remodeling (COLIA1, RANK, OPG, MMP2, OPN)

In 2011, Katz et al. conducted a cohort study on 78 MM patients taking intravenous BP therapy [46]. This paper reported that, over the 1-year study period, 12 patients developed BRONJ. The authors compared ten SNPs in seven genes (*CYP2C8*, *COL1A1*, *RANK*, *OPN*, *MMP2*, *OPG*, and *TNF*) in a candidate-gene study style. In this study, a combined genotype score of five SNPs (*COL1A1* rs1800012, *RANK* rs12458117, *MMP2* rs243865, *OPN* rs11730582, and *OPG* rs2073618) was able to significantly predict an 11-fold increase in MRONJ risk with a cutoff score of 5 [46].

*COL1A1* encodes an important part of type 1 collagen and its mutations are associated with osteogenesis imperfecta [75]. *COL1A1* rs1800012 may also be associated with ligament and tendon injuries [76]. RANK (receptor activator of the NF-kB) is a transmembrane protein expressed on osteoclasts and several other cells. It functions as the receptor of RANKL, and its activation is very important in osteoclast differentiation, activation, and survival [77]. Another very important molecule involved in osteoclastogenesis is osteoprotegerin (OPG), which functions as a decay receptor of RANKL [78]. *RANK* and *OPG* genetic polymorphisms have been previously linked to BMD [79]. *MMP2* encodes an important matrix metalloproteinase (matrix metallopeptidase 2, MMP2) that is involved in the cleavage of several extracellular and non-extracellular matrix molecules [80]. *MMP2* has previously been suggested as a candidate gene in MRONJ, as BPs are associated with atrial fibrillation, and MMP2 is associated with both bone and cardiovascular abnormalities [81].

These results support the role of bone remodeling abnormalities as a key factor in the pathogenesis of MRONJ and also suggest that genetic testing might be an effective tool for risk screening in the future. However, the small sample size poses a major limitation when interpreting these findings.

### 4.5. VEGFA

As abnormal and inhibited angiogenesis is a leading hypothesis in the possible pathophysiological mechanisms, it is logical that multiple papers have reported on SNPs in the *VEGFA* gene in MRONJ patients. *VEGFA* encodes the vascular endothelial growth factor (VEGF) protein, a key regulator of physiological (e.g., embryonic development) and pathological (e.g., solid tumors or intraocular neovascular syndromes) angiogenesis [82]. Moreover, VEGF is also an important molecule in inflammatory disorders and wound healing [82], and plays a crucial role in bone angiogenesis [83]. *VEGFA* polymorphisms have been linked to several diseases such as diabetic retinopathy, age-related macular degeneration, and different solid tumors [84,85,86,87]. VEGF also plays an important role in skeletal development [88]. Furthermore, postnatally osteoblast-derived VEGF regulates osteoblastogenesis and adipogenesis in bone marrow by stimulating RUNX2 and repressing PPARG2 [88]. It is hypothesized that polymorphisms associated with lower VEGF expression might have a pathophysiological role in the development of the condition.

In 2011, Arduino et al. were the first to analyze *VEGFA* polymorphisms in Italian female breast cancer patients with MRONJ [89]. The authors analyzed three SNPs (rs3025039, +936 C>T; rs699947, −2578 C>A; rs2010963, −634 G>C) in the *VEGFA* gene. The combined haplotype of CAC (+936/−2578/−634) was associated with MRONJ susceptibility [89]. All three of these polymorphisms have previously been reported to correlate with *VEGFA* expression [90,91,92], with two of them being associated with a lower expression level of VEGF (−2578/−634), and one of them with a higher expression level (+936). Analyzing only the two SNPs (−2578/−634) resulting in lower expression levels remained significantly associated with a higher incidence of MRONJ [89]. Another study conducted in the Korean population also evaluated these SNPs (rs3025039, rs699947, rs2010963), and further affirmed the association between *VEGFA* and the disease, finding that the CC genotype of both rs3025039 and rs2010963 was associated with a higher chance of developing the condition [93]. These results are partially consistent with a metanalysis in both polymorphisms of allele C being associated with MRONJ susceptibility, but not entirely identical, as there were some differences in the allele frequencies and the significant polymorphisms between the two studies, likely due to the different populations (Italian vs. Korean) studied. However, a more recent case–control study involving osteoporosis patients exclusively found no significant associations between rs2010963, rs3025039, and rs699947 and disease susceptibility [94]. On the other hand, two SNPs (rs881858 and rs10434) were found to be associated with MRONJ. The authors argue that rs10434 might also have an effect on VEGF expression based on different alleles being associated with different diseases, however, there is no direct proof of this in the literature [94]. A meta-analysis from 2020 involving 105 MRONJ cases found that rs3025039 was significantly associated with a risk for the condition [14].

Overall, there are some contradictions in the results considering *VEGFA* in MRONJ, which could have been caused by the different populations and different patients (e.g., osteoporosis vs. cancer patients) screened in the different studies. However, polymorphisms in *VEGFA*, which are associated with a lower level of VEGF expression, might pose a promising target for future investigation. Table 1 summarizes the studies investigating SNPs in the *CYP2C8*, *SIRT1*, and *VEGF* genes, which are among the most studied genes in MRONJ.

### 4.6. Other Genes Researched in MRONJ

In a 2022 study, 24 potentially pathogenic variants were identified using whole exome sequencing, with allele frequencies significantly different between the MRONJ-affected and control groups [95]. Nine of the polymorphisms clustered in only two genes. These genes are *KRT18* and *PABPC3*. The *KRT18* gene encodes a type 1 intermediate filament protein called keratin 18 [95]. It is interesting to note that the role of genes encoding other keratin proteins has been suggested in oral mucosal diseases [96]. Furthermore, KRT18 plays an important role in cytoskeleton organization and in various estrogen signaling pathways [95]. Malfunction of the KRT18 protein can lead to cytoskeletal dysfunction of the oral mucosal cells, which may predispose one to oral mucosal diseases and MRONJ. The *PABPC3* gene encodes a poly-A binding protein, whose functional role in the pathogenesis of the disease needs to be elucidated by further experimental and clinical studies.

A genome-wide association study identified a polymorphism (rs17024608) in the *RBMS3* gene, which was significantly associated with BRONJ incidence [97]. Variations of this gene have previously been linked to bone mass and osteoporotic fracture risk [97].

In another study, the *PPARG* (peroxisome proliferator-activated receptor gamma) SNP rs1152003 was also found to be associated with MRONJ in MM patients as well as SNPs in *ABP1* (amiloride binding protein 1), *CHST11* (carbohydrate sulfotransferase 11), and *CROT* (carnitine O-octanoyltransferase) genes [98]. *PPARG* polymorphisms have also been associated with bone remodeling and bone mineral density [98]. Interestingly, a study from 2017 found that the high-risk allele of *PPARG* rs1152003, together with *CYP2C8* rs1934951, was associated with early-onset of the disease [49]. Thus, these SNPs might be useful to assess the risk of early MRONJ development. Moreover, Poznak et al. found that *PPARG* rs1152003 had a significant association with MRONJ risk in an univariate analysis, however, after covariate adjustment, this SNP did not remain significantly associated [99].

A case–control study identified *farnesyl pyrophosphate synthase* (*FDPS*) rs2297480 allele A to be associated with MRONJ susceptibility [100]. FDPS functions as an important enzyme of the mevalonate pathway, and it is targeted by several amino-bisphosphonates [101,102]. Interestingly, *FDPS* rs2297480 allele A was also detected to be associated with an increased response to long-term amino-bisphosphonate therapy [102].

SNPs in genes encoding interleukins 1A and 1B have also been suggested to play a potential role in the pathogenesis of the disease [103].

**Figure 2:**
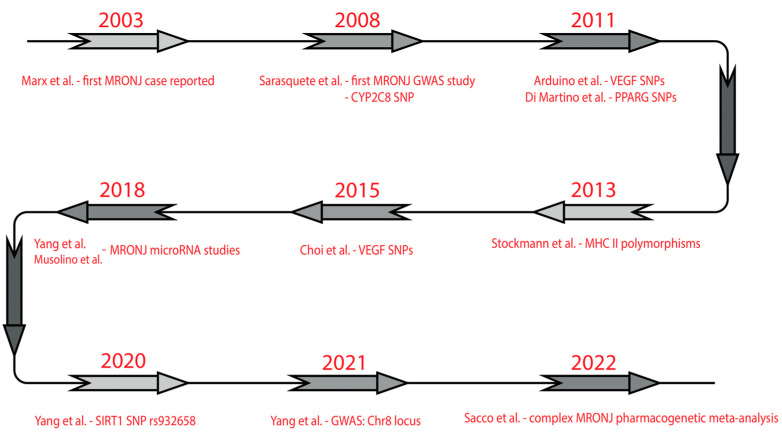
Timeline of some studies on the genetic background of MRONJ. Marx et al. [6], Sarasquete et al. [44], Arduino et al. [89], Di Martino et al. [98], Stockmann et al. [104], Choi et al. [93], Yang et al. [16,57,58], Musolino et al. [15], Sacco et al. [106].

Infection and immune system dysfunction are regularly mentioned as a major morbidity factor in MRONJ. In a case–control study of 204 patients, several variants of Human Leukocyte Antigen Class II (HLA Class II) (DRB1*15, DQB1*06:02, DRB1*01, and DQB1*05:01) were significantly more prevalent in MRONJ patients [104]. These HLA antigens are involved in immune cell differentiation and antigen presentation [104]. Figure 2 highlights some studies on the genetic background of the disease.

**Table 1:** Summary of studies investigating SNPs of the *CYP2C8*, *SIRT1*, and *VEGF* genes in MRONJ.

SNP	Study	Location	Study Design	Sample Size (Cases/Controls)	Study Population	Drug Administered
*CYP2C8* rs1934951 rs1934980 rs1341162 rs17110453	Sarasquete et al. [44]	Spain	GWAS	22/65	MM patients	Zoledronic acid, pamidronate
*CYP2C8* rs1934951	English et al. [45]	USA	Candidate gene study	17/83	Advanced prostate cancer patients	Zoledronic acid, alendronate, pamidronate
*CYP2C8* rs1934951	Such et al. [47]	Spain, Greece	Candidate gene study	42/37 + 45 ^1^	MM patients	Zoledronic acid
*CYP2C8* rs1934951	Katz et al. [46]	USA	Candidate gene study	12/66	MM patients	Pamidronate, zoledronic acid
*CYP2C8* rs1934951	Balla et al. [48]	Hungary	Candidate gene study	46/224	Osteoporosis, breast and other cancers	Alendronate, pamidronate, zoledronate, ibandronate, rizedronate, clodronate
*CYP2C8* rs1934951	Kastritis et al. [49]	Greece	Candidate gene study	36/104	MM patients	Zoledronic acid
*CYP2C8* rs1934951	Poznak et al. [99]	USA	Candidate gene study	76/126	MM, breast, prostate, and other cancers	Zoledronic acid, pamidronate
*SIRT1* rs7896005	Yang et al. [57]	USA, Hungary, Italy	WES	22/22	MM patients	Zoledronate, pamidronate
*SIRT1* rs7896005 rs375839 rs932658 rs2394443	Yang et al. [58]	USA, Hungary, Italy	Candidate gene study	46/58	MM, breast and other cancers	-
*SIRT1* rs7894483 rs7896005 rs3758391 rs932658	Bojtor et al. [59]	Hungary	Candidate gene study	63/0	MM, osteoporosis, cancers	BPs, denosumab
*VEGFA* rs699947 rs2010963 rs302503	Arduino et al. [89]	Italy	Candidate gene study	30/30	MM, breast cancer	Zoledronic acid
*VEGFA* rs699947 rs2010963 rs302503	Choi et al. [93]	Korea	Candidate gene study	26/19	Osteoporosis,	Alendronate, ibandronate, rizedronate, zoledronic acid
*VEGFA* rs202125661	Lee et al. [105]	Korea	WES	38/90	Osteoporosis, cancers	BPs
*VEGFA* rs2010963 rs699947 rs10434 rs25648 rs3024987 rs3025022 rs3025035 rs3025039 rs998584 rs6905288 rs881858	Kim et al. [94]	Korea	Candidate gene study	58/67	Osteoporosis	BPs
*VEGFA* rs833061 rs699947 rs2010963 *VEGFC* rs7664413 rs2333496 rs6838834 rs3775203	Poznak et al. [99]	USA	Candidate gene study	76/126	MM, breast, prostate, and other cancers	Zoledronic acid, pamidronate

^1^ Number of controls plus number of healthy individuals.

## 5. Gene Expression Studies in MRONJ

By analyzing the mRNA pattern of MRONJ tissue samples, we can obtain a snapshot of its gene expression pattern. Such studies will not only provide a better understanding of the pathomechanism of the disease, but also identify potential drug targets.

One study found differential expression of defensins, a family of antimicrobial proteins, in MRONJ bone tissue samples [18]. Two types of defensin molecules (DEFA3, DEFB3) were also more highly expressed in denosumab-induced osteonecrosis of the jaw than in the control tissues [18]. This result further confirms the role of chronic infection in the pathophysiology of the disease.

The expression of factors promoting vascular remodeling was also investigated in a rat model [107]. Increased expression of factors promoting angiogenesis (e.g., VEGFA, VEGFR-2) was observed in this experiment. However, it has been suggested that a compensatory mechanism due to the inhibitory effect of zoledronic acid on vascularization may underlie this. Further studies with a larger number of human cases are needed to explain this precisely.

An in vitro study demonstrated that low-dose BPs such as zoledronate, alendronate, and clodronate could alter the expression of vital genes (e.g., *TGF-B1*, *TGF-BR1*, *TGF-BR2*, *RUNX-2*, *Col-1*) in osteoblast growth and differentiation and osteoblast–osteoclast interactions [108]. Wehrhan et al. found that Msx-1, an important transcription factor promoting cellular plasticity and differentiation in the periodontal ligaments, was significantly underexpressed in BRONJ tissue [109]. The underexpression of Msx-1 can explain the common clinical finding of sclerotic remodeling of the periodontal ligaments in MRONJ cases [109].

By examining the transcriptional pattern of genes involved in immunological processes, the locally altered immune environment of MRONJ tissue can be determined [19]. Moreover, individuals who were more susceptible to BRONJ had lower gene expression levels for several important factors (RANK, AHR, FGF9), determining immune resilience and normal barrier function [110]. In these individuals, the added stress of BP administration might cause BRONJ development [110]. A bioinformatic analysis, conducted on samples from MM patients with or without BRONJ, revealed that differently expressed genes (DEGs) in BRONJ cases were mainly enriched in pathways that are important in immune functions and RNA splicing such as TNF, ILB1, and DDX5 [111,112].

In conclusion, gene expression studies can provide information on the current state of the transcriptome of tissue affected by osteonecrosis of the jaw, which may provide valuable insights into the pathomechanism of MRONJ, and may also identify potential new therapeutic targets. Moreover, the evaluation of combined gene expression panels might provide new biomarkers in the disease [113]. Table 2 summarizes studies analyzing gene expression in connection with MRONJ.

**Table 2:** Summary of gene expression studies in MRONJ.

Gene	Study	Study Design	Sample	Sample Size (Cases/Controls)	Overexpressed Gene(s)	Under Expressed Gene(s)
*Msx-1*, *RANKL*, *BMP-2/4*	Wehrhan et al. [109]	Case–control study	Human oral mucoperiosteal specimens	20/20	BMP-2/4	Msx-1 RANKL
54600 genes by the Affymetrix U133Plus 2.0 Gene Chip	Raje et al. [112]	Case–control study	Human peripheral mononuclear blood cells	11/10/5	Osteoclast-inhibiting factors	Genes involved in osteoblast signaling, differentiation, and activation
*FGF9*, *IFNG*, *TNFA*, *IL1B*, *IL17*, *CTGF*, *RANK*, *RANKL*, *MMP7*, *MMP9*, *GMCSF*, *AHR*	Kalyan et al. [110]	Case–control study	Human peripheral blood samples	6/87	-	RANK, RANKL, TNFA, FGF9, GMCSF, CTGF, MMP-7, AHR
*Runx-2, OSX, ALP, OSC, OPG, RANKL, Col-I, BMP-2, BMP-7, TGF-β1, VEGF, TGF-βR1, TGF-βR2, TGF-βR3*	Manzano-Moreno et al. [108]	In vitro study analyzing bisphosphonate modulation of the gene expression	HOBS and MG-63 osteoblast cell lines	-	Osteoblast growth factors (TGF-β1, TGF-βR1, TGF-βR2, TGF-βR3, and VEGF)	Cell maturation factors (RUNX-2, Col-1, OSX, OSC, BMP-2, BMP-7, or ALP)
*hAD-1*, *hAD-3*, *hBD-1*, *hBD-3*	Thiel et al. [18]	Case–control study	Human bone specimens	12/6	hAD-3, hBD-3	-
Affymetrix Gene Expression Array	Shi et al. [114]	In vitro study analyzing zoledronate modulation of the gene expression	Human PDLSCs	-	Genes associated with cellular stress response signaling pathways	Genes associated with proliferation- and ossification-associated signaling pathways
*CCR7*, *F4/80*, *CD206*, *cxcl12*, *cxcr4*, *CD105*, *IL-6*, *TNF-α*, *IL-1β*, *IL-10*, *TGF-β*, *IGF-1*, *VEGFA*, *VEGFB*, *VEGFC*	Kuroshima et al. [115]	BRONJ mouse model	Extraction wound soft tissue samples from female C57BL/6J mice	-	Inflammatory cytokine-related genes	Anti-inflammatory cytokine-related and wound enhancement related genes

## 6. MicroRNAs and Other Noncoding RNAs in MRONJ

MicroRNAs (miRNAs) are small noncoding RNA molecules that play a crucial role in the regulation of gene expression at the messenger RNA (mRNA) level [116]. They primarily exert their repression of gene translation by selectively binding to the 3′UTR (untranslated region) of their target mRNA [117]. Altered miRNA expression patterns have been linked to several diseases including cardiovascular abnormalities (e.g., cardiac hypertrophy, cardiac failure), metabolic diseases (e.g., diabetes mellitus), and different cancers [116]. MiRNAs have also been described as crucial regulators in bone formation and homeostasis including osteogenesis, osteoclastogenesis, and the regulation of osteoblasts [17]. Due to their cardinal role in bone biology, some miRNAs have been examined in both human and animal MRONJ studies.

In 2018, Musolino et al. investigated 18 different miRNAs expressed in peripheral lymphocytes in 10 MM patients (5 cases and 5 controls) with or without MRONJ [15]. In this study, 14 miRNAs were found to have a significant difference in expression between the groups, all of them being overexpressed. Six of these miRNAs (miR-16-1, miR-149, miR-23-a, miR-145, miR-129-1, miR-221) displayed strong overexpression (4-fold to 11-fold) [15]. Notably, all of them regulate pathways that are important in bone homeostasis and inflammatory processes [15]. Another study compared 6 BRONJ cases and 11 healthy controls [16]. In this study, miR-21 and miR-23-a were found to be overexpressed, while miR-145 was found to be underexpressed in the sera of BRONJ patients [16].

There are also some preliminary data available regarding other noncoding RNAs that might be potential contributors to the disease. Allegra et al. found distinct expression profiles of long noncoding RNAs (lncRNAs) in MM patients with MRONJ compared with MRONJ-free MM patients [118].

These possible new findings might help better understand the pathophysiology of MRONJ as well as identify new targets for therapy. For example, the inhibition of miR-23-a improved femoral head osteonecrosis in rat models [119]. Another study found that the local administration of miR-149-5p loaded vesicles promoted better wound healing in a BRONJ mouse model [120]. Despite these promising results, further studies are needed to better understand the role of noncoding RNAs in MRONJ.

## 7. Conclusions and Future Perspectives

A review of the literature showed that several genes and gene variants have been implicated in the pathogenesis of MRONJ. The initial candidate gene studies have been complemented by whole exome and genome-wide sequence analyses with the introduction of high-throughput NGS technology, which has significantly expanded our knowledge of the complex genetic background of MRONJ. The role of some gene variants (e.g., *SIRT1*, *VEGF* in genes) has been confirmed by several independent studies. In addition, there are newly described potential genes that need further validation (e.g., *KRT18*), which may bring us closer to a deeper understanding of the background of MRONJ. The heterogeneous nature of MRONJ might complicate the identification of universal genetic factors, however, in the future, in addition to the methods presented in this summary, which primarily detect sequence divergences, for example, by epigenetic or DNA methylation or mRNA or microRNA expression studies, we may gain a more complete picture of the diverse genetic background of the disease.

MRONJ is a complex, multifactorial disease in which the genetic factors described above also play a key role. Despite the major negative impact of the disease on quality of life, there is currently no clinical risk assessment system that can provide a good approximation of which patients should be expected to develop this adverse side effect.

Several clinical trials have been conducted on patients with this condition to find novel therapeutical modalities to tackle this disease. For instance, a study found that teriparatide had a beneficial effect on bone healing in MRONJ without any additional adverse effects [121]. Another study detected that the addition of bone morphogenic protein-2 (BMP-2) to leukocyte-rich and platelet-rich fibrin improved postoperative outcomes in MRONJ patients [122]. The integration of emerging therapeutic strategies complemented by novel prognostic biomarkers can help further enhance the management of this condition.

Known risk factors, combined with newly identified genetic predisposing factors, could be used to develop a personalized algorithm including genetic diagnostics to screen patients at high risk for MRONJ. Greater therapeutic attention to them (e.g., performing necessary dental procedures prior to antiresorptive therapy) could reduce the incidence of osteonecrotic side effects when administering antiresorptive agents. This could improve the quality of life of patients as well as improve the success of the treatment of the often malignant underlying disease. This will require further large case–control studies in the future to confirm the role of known genetic factors and identify new genetic risk factors. The introduction of novel scientific approaches can help further improve the knowledge of MRONJ genetics. For instance, the founding of a biobank of MRONJ samples and related genetic data could facilitate large-scale studies on multi-centered study groups. Longitudinal genetic studies would also be beneficial to better understand the development of this disease. Furthermore, analyzing epigenomic data could help understand the effects of environmental factors on gene expression and genetic susceptibility. Finally, the implementation of polygenic risk scores (PRSs) based on GWAS data can provide an overview of the genetic effects in MRONJ by aggregating the combined effect of several lower-impact polymorphisms. By calculating the PRSs, high-risk individuals can be identified, which could lower the MRONJ incidence in patients at risk.

## Data Availability

No new data were created or analyzed in this study. Data sharing is not applicable to this article.
